# New trends and hotspots in sepsis-related protein post-translational modification: a bibliometric and visual analysis

**DOI:** 10.3389/fmed.2025.1606786

**Published:** 2025-07-22

**Authors:** Lin Song, JingYi Ma, Wei Jiang, Ke Liu, Jing Wang, Hua Lin, Jiangquan Yu, Ruiqiang Zheng

**Affiliations:** ^1^Northern Jiangsu People's Hospital Affiliated to Yangzhou University, Yangzhou, China; ^2^Intensive Care Unit, Northern Jiangsu People’s Hospital, Yangzhou, China; ^3^Yangzhou University Hospital, Yangzhou, China

**Keywords:** post-translational modification, bibliometric, ubiquitination, methylation, sepsis

## Abstract

**Background:**

Sepsis is a clinical syndrome characterized by high morbidity and mortality rates, posing a severe threat to human health. Its pathophysiology is complex, involving multiple physiological and pathological processes. Protein post-translational modification (PTM) play a pivotal role in the pathophysiology of sepsis by regulating inflammation, immune responses, and organ dysfunction. In recent years, there has been a growing focus on the association between sepsis and PTM; however, a comprehensive and systematic analysis of the current research status and development trends in this field is still lacking.

**Methods:**

This study analyzed literature from the Web of Science Core Collection published between 2005 and 2024. CiteSpace, VOSviewer, and Excel facilitated the bibliometric analysis, visualizing publication trends, contributions across countries/regions and institutions, journal distributions, author collaboration networks, and keyword clusters.

**Results:**

A total of 1705 articles were included, originating from 58 countries/regions. The annual publication volume showed exponential growth (*R*^2^ = 0.9662), with China leading the way (48.68%), followed by the United States (29.27%). Shanghai Jiao Tong University emerged as a high-yield institution (*n* = 51), while the University of Pittsburgh demonstrated the highest citation impact (with an average of 109.87 citations per article). Prominent journals featuring these articles include Shock (*n* = 77) and the Journal of Immunology (with an average citation of 65.75 times per article). Research hotspots were centered around phosphorylation, ubiquitination, and methylation, with emerging trends such as sepsis-associated acute kidney injury (SA-AKI), autophagy, and mitochondrial dysfunction.

**Conclusion:**

Research on the sepsis-related PTM is flourishing. This study systematically reveals the research dynamics and core trends in this field.

## Graphical abstract

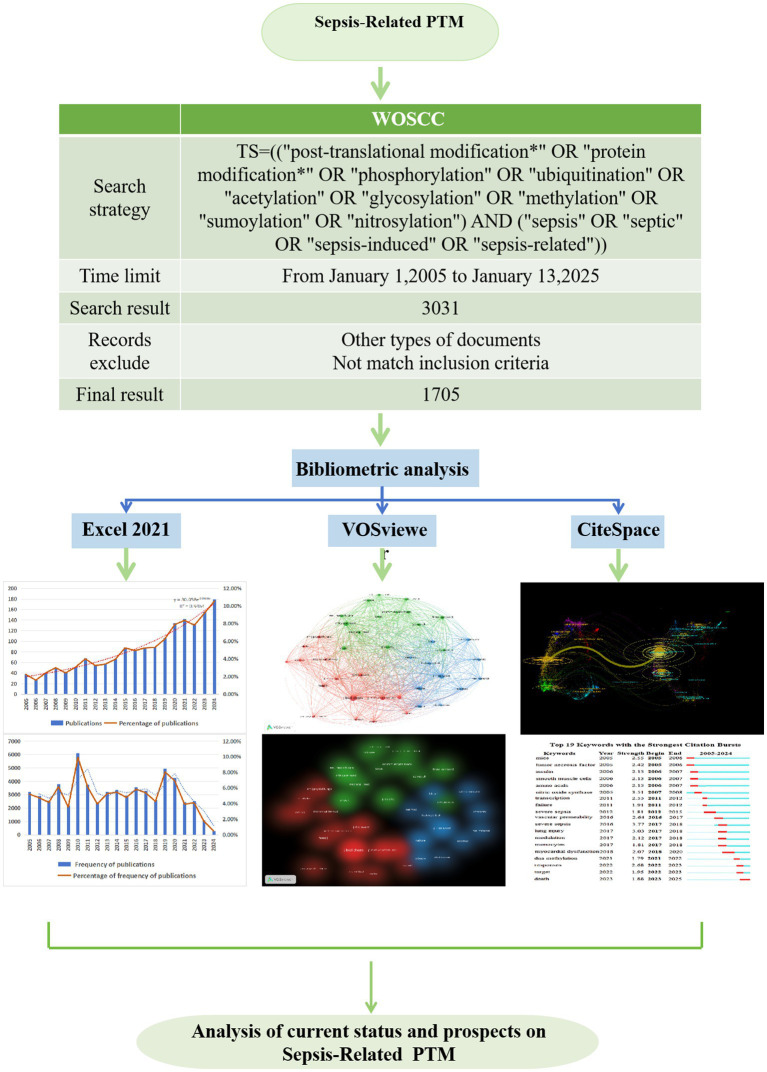

## 1 Introduction

Sepsis, a life-threatening organ dysfunction caused by dysregulated host responses to infection (1), remains a critical global health challenge. There were approximately 48.9 million cases of sepsis worldwide, with 11 million related deaths reported, accounting for 20% of global deaths (2). Moreover, even among discharged patients, 40% of those with severe sepsis are re-admitted within 90 days, and only 20.5% of severe sepsis survivors remain alive and out of hospital for 1 year without re-admission (3). This undoubtedly increases the burden on individual patients, as well as on the healthcare system and society. Since the launch of the “Surviving Sepsis Campaign” in 2002, clinical practices such as early fluid resuscitation, antimicrobial therapy, and organ support have been standardized, leading to some improvements. However, patient mortality rates remain stubbornly high (4). Therefore, comprehensively understanding the pathophysiological changes during the development of sepsis and exploring new diagnostic markers and therapeutic targets have become a research hotspot and focus in the current medical field.

Post-translational modification (PTM) comprise covalent alterations of proteins following RNA translation, forming a crucial phase in protein biosynthesis (5). These modifications substantially influence sepsis pathophysiology by modulating inflammatory responses, immune regulation, and metabolic reprogramming (6). Among PTM, phosphorylation dominates current research due to its central role in sepsis-related inflammatory signaling pathways. During LPS-induced septic shock, Pin1 mediates p38 MAPK phosphorylation, thereby activating NLRP3 inflammasome-dependent pyroptosis (7). Lactylation, a newly discovered PTM, also plays a significant role in the metabolic and immune regulation of sepsis. It promotes the cytoplasmic localization and exosome release of High Mobility Group Box 1 (HMGB1), exacerbating the inflammatory response and organ damage in sepsis (8). Furthermore, PTM-related products have great potential in the diagnosis and treatment of sepsis. Citrullinated Histone H3 (CitH3) (9, 10) and lactylated Histone H3K18 (11) can serve as potential biomarkers for diagnosing and predicting the severity of septic shock. In an LPS-induced AKI mouse model, the selective class IIa HDAC inhibitor (HDACi) TMP195 demonstrates potent renal protective effects in SA-AKI by reducing renal tubular cell apoptosis and inflammation (12). Mounting evidence suggests that PTMs may be the cornerstone of regulating cellular functions and various diseases, including sepsis. However, it remains incompletely understood how infections induce specific protein PTM and how these modifications subsequently lead to inflammatory diseases, cytokine storms, disseminated intravascular coagulation, and organ dysfunction (13).

Growing evidence links sepsis to PTM, yet no bibliometric analysis has systematically examined this relationship. Earlier reviews adopted qualitative approaches, synthesizing findings through narrative summaries rather than quantitative methods. However, bibliometrics employs quantitative analysis of literature data to visually present influential authors, institutions, countries, high-frequency keywords, citation networks, and trending topics through charts and visual graphics. This approach facilitates the evaluation of academic impact and quality while providing valuable guidance for scientific research (14, 15). Therefore, this study aims to conduct a bibliometric analysis of research on protein post-translational modifications (PTMs) in sepsis from 2005 to 2024. Specifically, we will (1) identify publication trends and core contributors in this field, (2) explore major research themes, analyze emerging trends and potential research directions. By addressing gaps in existing literature, this study seeks to provide a systematic overview of the current state of the field, support the scientific integration of PTM with sepsis pathophysiology, and offer a theoretical foundation for developing novel therapeutic strategies.

## 2 Materials and methods

### 2.1 Data source and retrieval

**Figure 1:**
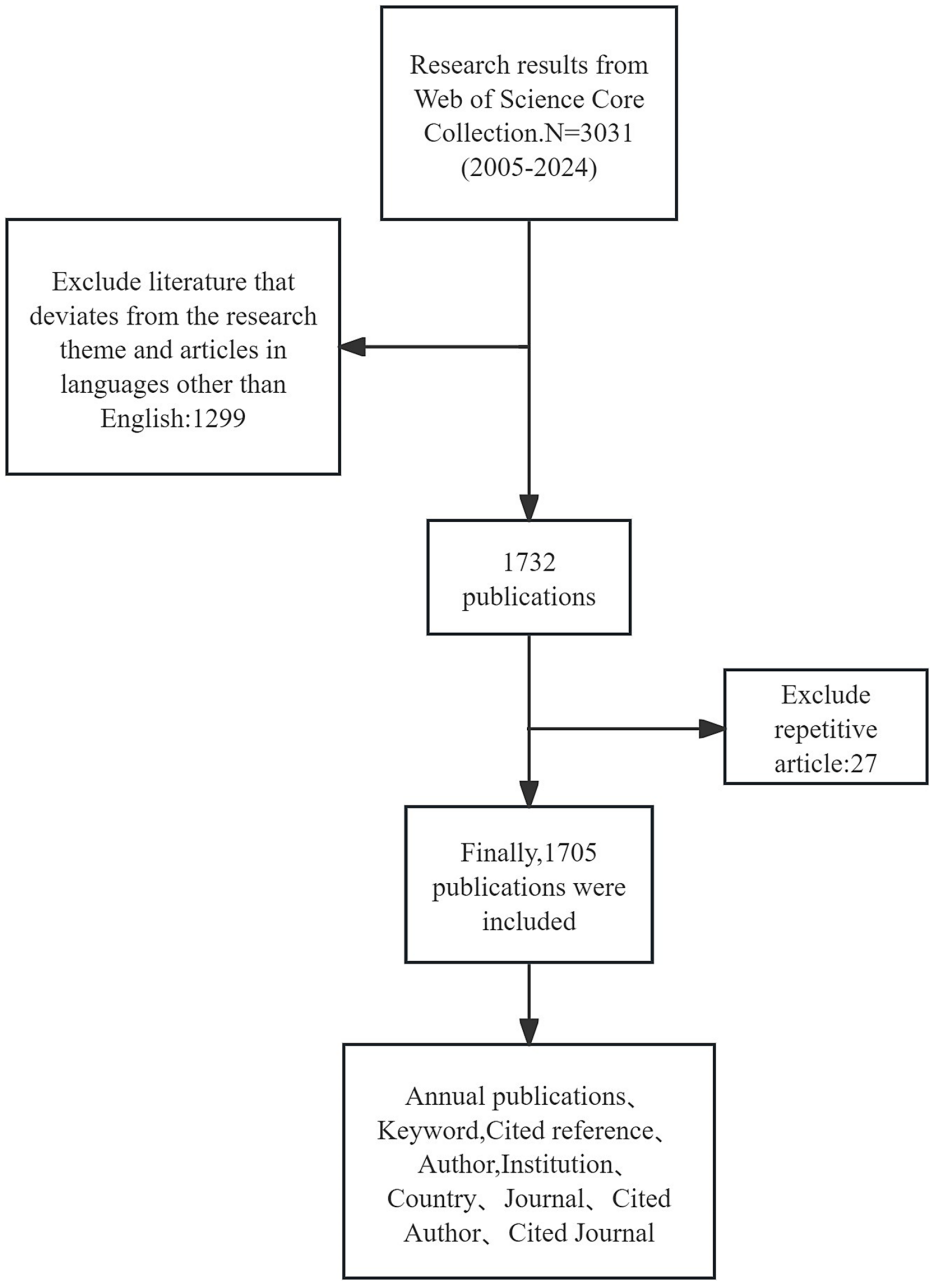
Flowchart of the literature search and selection process.

This study selected the Science Citation Index and Social Citation Index from the Web of Science Core Collection as data sources. The search query used was TS = ((“post-translational modification*” OR “protein modification*” OR “phosphorylation” OR “ubiquitination” OR “acetylation” OR “glycosylation” OR “methylation” OR “sumoylation” OR “nitrosylation”) AND (“sepsis” OR “septic” OR “sepsis-induced” OR “sepsis-related”)), with a time range from January 2005 to December 2024. The article types chosen were articles and review articles, written in English, resulting in a total of 3,031 retrieved articles. Two evaluators independently screened the retrieved literature, removing duplicates, articles that did not meet the search criteria or deviated from the research topic, and articles in languages other than English. Following discussion and consensus, a total of 1705 valid articles were identified. This process is illustrated in Figure 1. The downloaded data included titles, authors, publication years, countries/regions, institutions, keywords, abstracts, references, etc., and the documents were downloaded in plain text format with tabular delimiters.

### 2.2 Data analysis and visualization

All raw data were extracted from the WoSCC database, and the following bibliometric analysis was conducted using the software applications: CiteSpace, VOSviewer, and Microsoft Excel 2021.

CiteSpace is a complex visualization tool designed to analyze and visualize scientific literature. By mapping citation networks, identifying key authors, pivotal articles, emerging trends, and influential research clusters, it aids researchers in exploring the knowledge structure of specific domains. Through CiteSpace, we were able to perform deduplication, cluster analysis, and timeline view analysis of the literature. Additionally, we conducted a time series analysis of keywords and generated visual representations with the following parameters: time span: 2000–2021, slice year: 1; selection criteria Top 10%, No pruning mode. This allowed us to discover the process of knowledge advancement over time and understand the developmental progress and trends in the field of post-translational modification of proteins and sepsis.

VOSviewer is software used to construct and visualize bibliometric networks. It excels in creating maps based on citation, co-authorship, and co-occurrence data, enabling researchers to gain insights into the relationships and patterns within scientific literature. VOSviewer simplifies the process of analyzing large datasets and presents results through visually appealing graphical representations. In this paper, VOSviewer was utilized to analyze the relationships between countries/regions, institutions, and authors, as well as to conduct cluster analysis on keywords and research hotspots. The parameters were set as follows: Country: minimum number of citations of a country: 0, minimum number of documents of a country: 5; Institutions: minimum number of citations of an organization: 0, minimum number of documents of an organization: 5; Source: minimum number of documents of a source: 8, minimum number of citations of a source: 0; Author: minimum number of citations of an author: 0, minimum number of documents of an author: 5; Citations of a source: minimum number of citations of a source: 361; Citations of a reference: minimum number of citations of a reference: 25; Keywords: minimum number of occurrences of a keyword: 47.

Microsoft Excel 2021 was used to analyze and plot the annual publication volume, as well as the quantity and trend charts of citation frequency. This facilitated a comprehensive observation of trends over the years and allowed for predictions of future directions and trends.

## 3 Results

### 3.1 The annual trend of paper publication quantity and citation times

**Figure 2:**
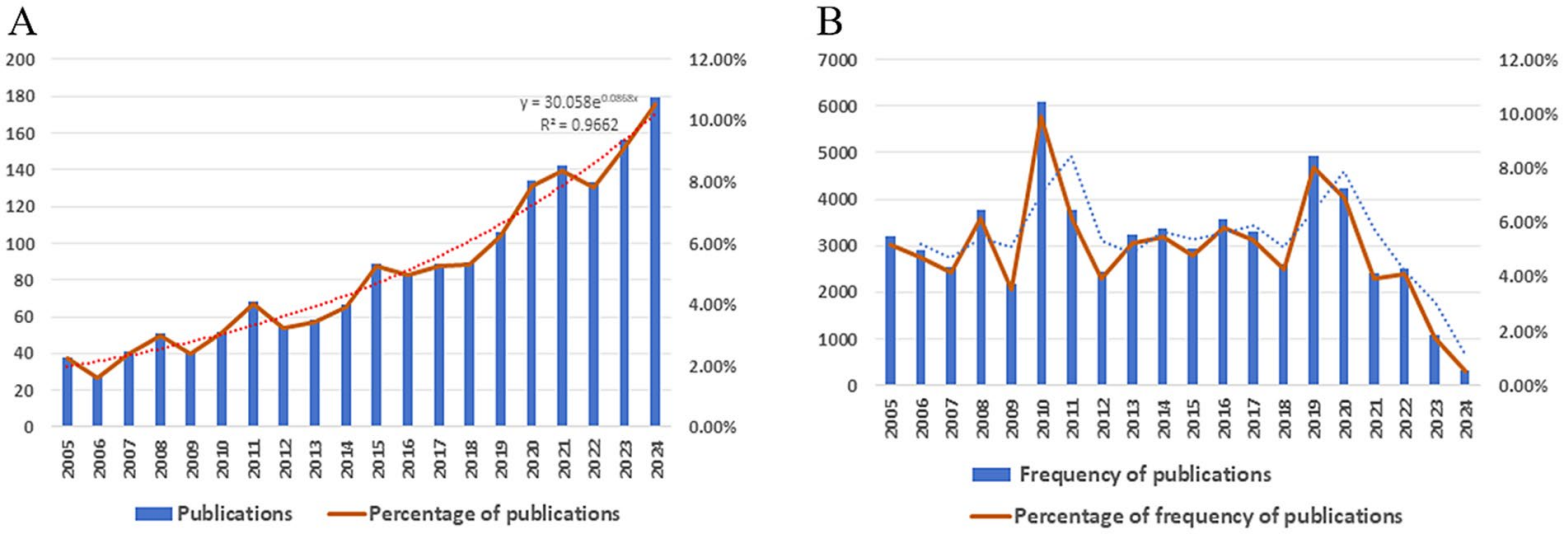
**(A)** Trends in publication volume on post-translational modifications and sepsis (2005–2024). **(B)** Temporal trends in citation frequency of publications examining PTM and sepsis (2005–2024).

This study analyzed 1705 publications on protein PTM and sepsis from 2005 to 2024, sourced from the Web of Science Core Collection’s Science Citation Index and Social Sciences Citation Index. Articles constituted 94.19% (1606) of the corpus, while review articles accounted for 5.81% (99) (Figure 2A). Publication output showed consistent growth over two decades, with a particularly sharp rise from 106 to 134 papers between 2019 and 2020. By 2024, annual publications had quintupled compared to 2005 levels, with exponential fitting demonstrating a strong temporal correlation (*R*^2^ = 0.9662). Although citation rates remained generally stable (Figure 2B), publications from 2010 and 2019 received disproportionately high citation counts, reflecting their substantial field influence. These citation patterns suggest that examining the methodologies and findings of these impactful studies could guide future research directions. The highly cited works likely focus on specific PTM types (e.g., phosphorylation or ubiquitination) and their mechanistic roles in sepsis pathophysiology, potentially including novel diagnostic markers or therapeutic targets. Systematic analysis of these seminal papers may help identify promising avenues for advancing the field.

### 3.2 Analysis of top productive countries/regions

**Figure 3:**
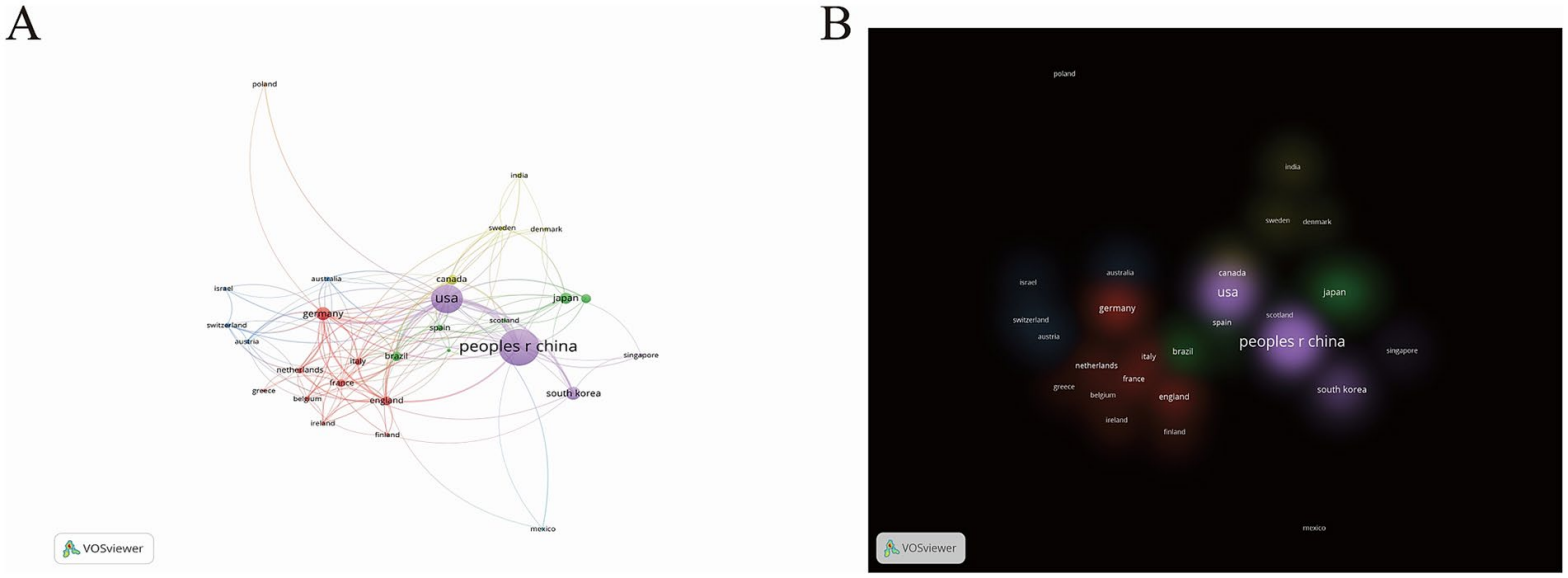
**(A)** The citation network visualization map of Nation/Region. **(B)** The citation density visualization map of Nation/Region. In network visualization map, nodes in distinct hues denote respective clusters; node dimensions correlate with citation frequency, where larger sizes signify greater influence and dominance; edge thickness reflects the strength of co-citation linkages between journals, with bolder lines indicating tighter relationships. In the density map, luminous zones represent elevated density, while subdued areas indicate diminished density.

The analysis then examined the geographical distribution of publications across countries. Among the 58 countries/regions represented in this study, China contributed the most articles (830; 48.68%), far exceeding other nations. The United States ranked second with 499 publications (29.27%), while South Korea and Germany shared third place with 104 articles each (6.10%) (Supplementary Table 1). These disparities likely stem from variations in national research funding, infrastructure, and workforce capacity. To visualize international collaborations, we generated a country/region network map with VOSviewer (Figure 3A), where line thickness corresponds to collaboration frequency. China, the United States, and Korea exhibited particularly strong partnerships, whereas Germany primarily collaborated with European nations like the UK and France. The United States, China, and Germany demonstrated the highest total link strength, reflecting their central roles in this research domain. This mode of international collaboration fosters knowledge sharing, resource integration, and the rapid transformation of research outcomes. Figure 3B illustrates a country/region distribution map based on cluster density analysis. A purple cluster centered around China occupies a significant weight, indicating China’s strong research capability and clustering effect in this field, driving the common development of surrounding countries and regions. Additionally, a red cluster led by Germany demonstrates high research activity, suggesting that Europe also possesses significant research strength in this area.

The formation of these clusters may be closely related to factors such as national scientific research policies, the layout of research institutions, and disciplinary development directions. Future studies could further analyze research themes and collaboration modes within these clusters to gain a deeper understanding of the research characteristics and advantages of different regions. The current geographical distribution provides valuable insights for future international collaboration and resource allocation. Further research can delve into the factors influencing this distribution and offer strategic suggestions to promote balanced development in global sepsis and PTM research.

### 3.3 Analysis of top productive institutions

**Figure 4:**
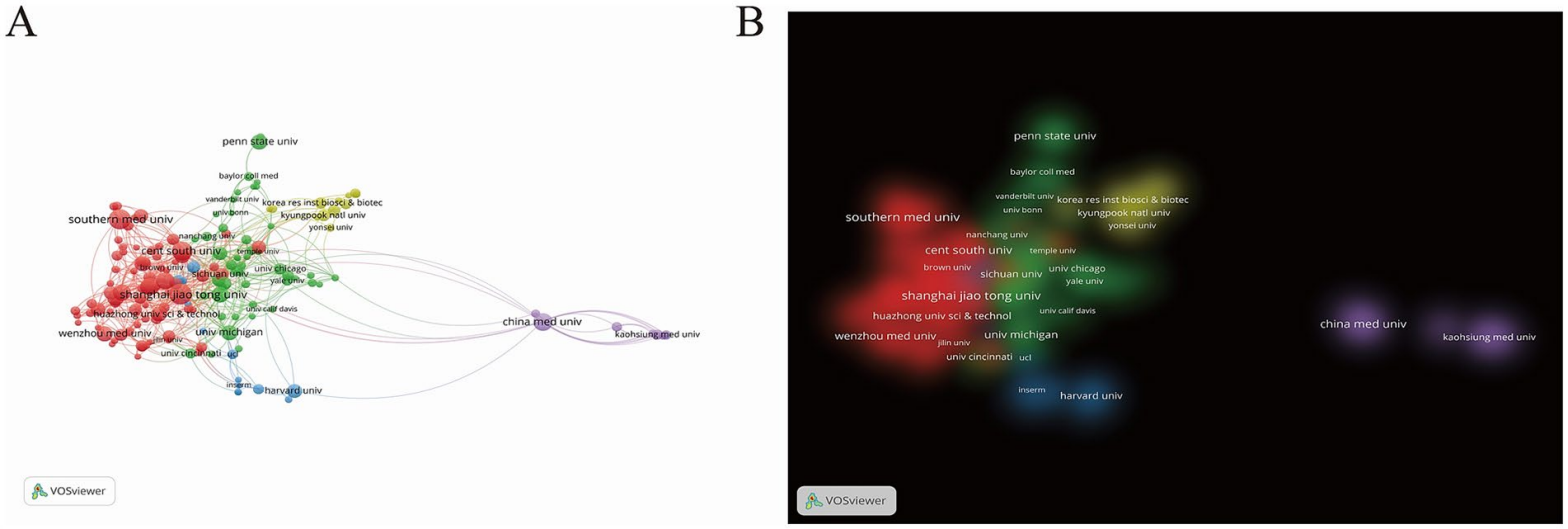
**(A)** The citation network visualization map of institutions. **(B)** The citation density visualization map of institutions.

Visual analysis of institutional collaboration networks with VOSviewer (Figures 4A,B) identified 167 institutions that published ≥10 papers among the 1807 analyzed, forming five distinct research clusters with defined thematic orientations (institutions sharing colors exhibit thematic similarities and strong collaborations). The red cluster, anchored by Shanghai Jiao Tong University, primarily investigates PTM’s involvement in sepsis-associated cell death, inflammatory responses, organ dysfunction, metabolic regulation, and immune modulation, with collaborative ties extending across Chinese medical institutions. Other clusters incorporate partners like the University of Pittsburgh and Kyungpook National University, demonstrating how interregional collaborations drive progress in sepsis-related PTM research. The ten most productive institutions have published 364 papers collectively, accounting for 21.35% of the total output. Shanghai Jiao Tong University (*n* = 51, 2.99%), Southern Medical University (*n* = 49, 2.87%), and Central South University (*n* = 43, 2.52%) lead in publication numbers (Supplementary Table 2). Despite ranking second in output (*n* = 30, 1.76%), the University of Pittsburgh demonstrates substantially higher citation counts, reflecting its pivotal role in advancing the mechanistic understanding of PTM-sepsis interactions. Universities dominate the institutional landscape, with regional specialization evident among the top contributors: China (Shanghai Jiao Tong University) prioritizes molecular mechanisms, signaling pathways, and therapeutic targets in sepsis-associated PTM; the United States (University of Pittsburgh) emphasizes sepsis-induced acute kidney injury, metabolic dysregulation, and inflammasome pathways; and South Korea (Kyungpook National University) focuses on therapeutic interventions using specific compounds and natural products for inflammatory responses.

### 3.4 Journal distribution

**Figure 5:**
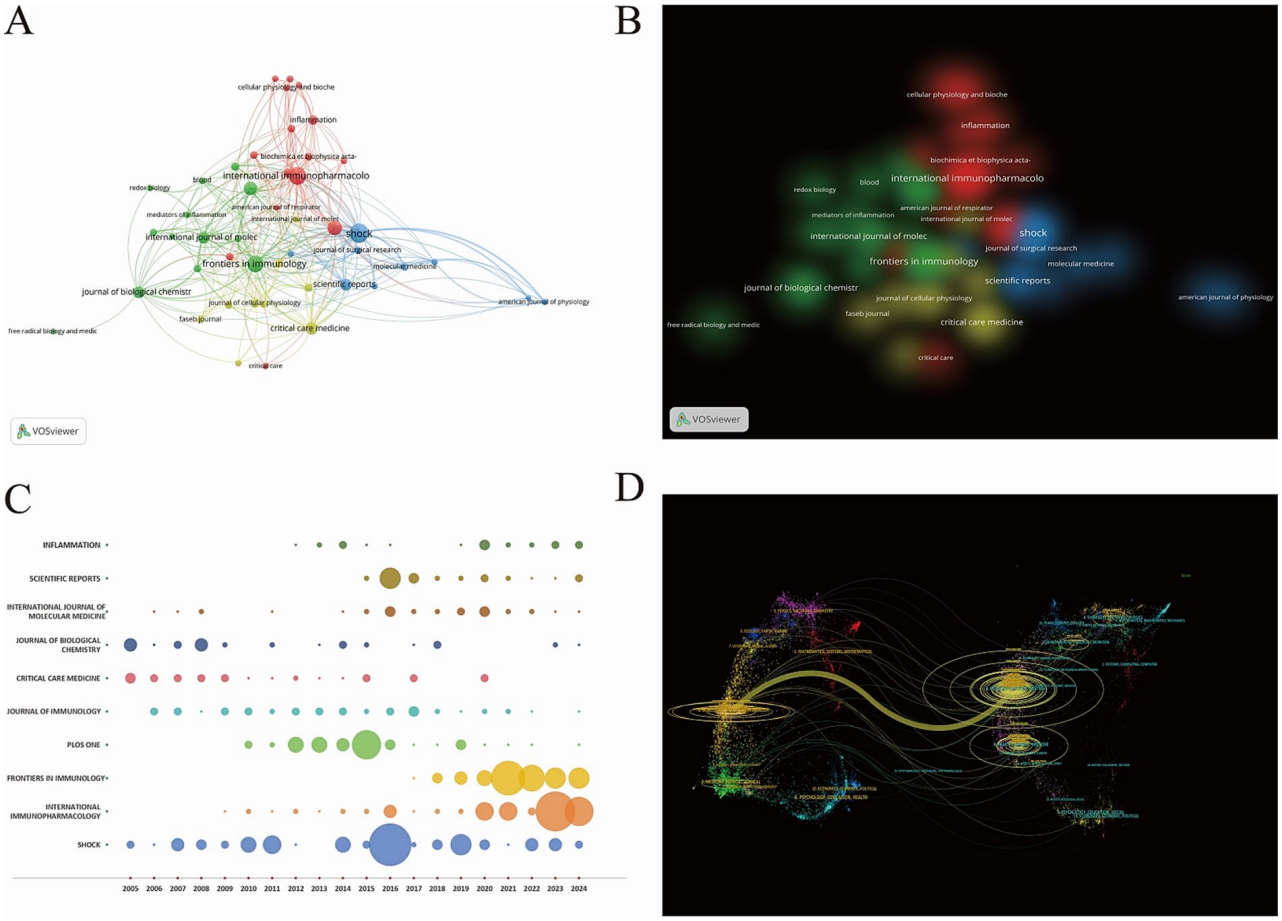
**(A)** Network visualization map of journal distribution. **(B)** Density visualization map of journal distribution. **(C)** Annual publication output of top-ten journals by year: bubble plot visualization (bubble area corresponds to publication volume: expansive scale = higher output). **(D)** A dual-map overlays on PTM and sepsis (the left diagram delineates the citing journals, while the right delineates the cited journals; distinct hues signify diverse thematic clusters, with connecting lines representing citation pathways between them. Thicker lines denote greater predominance in these scholarly interconnections).

The research papers exploring the relationship between PTM and sepsis exhibit significant diversity in their sources. According to statistics, a total of 502 journals have published relevant papers. The top ten journals by publication volume are listed in Supplementary Table 3, among which “Shock” has the highest number of publications, ranking first (*n* = 77, 4.51%). Meanwhile, the “Journal of Immunology” has the highest average citation rate (averaging 65.75 citations), making it the most influential journal in this field. Analysis of the articles published in the “Journal of Immunology” reveals that they primarily consist of original research papers and reviews. The research focus is concentrated on the role of PTMs in the pathophysiology of sepsis, the potential of PTM as biomarkers for the diagnosis and prognosis of sepsis, and the possibility of PTM serving as potential therapeutic targets for sepsis. Using VOSviewer, a visual map of journal distribution was constructed, as shown in Figures 5A,B. The journals can be roughly divided into four clusters, with journals in the same cluster publishing articles with similar research directions and focuses. These four clusters have similar weights and are all core journals with high publication and citation volumes in the field of PTM and sepsis. As illustrated in Figure 5C, the number of publications in immunology-related journals in this field has been increasing in the past 5 years. The dual-map overlay of the journals illustrates the thematic distribution of the publications. The citing journals are located on the left side of the map, while the cited journals are on the right. The colored paths represent the relevance of references and the flow and connection of knowledge from different research fields. A prominent citation path has been identified. The yellow path indicates that research published in Molecular Biology and Immunology is frequently cited in studies published in Molecular Biology and Genetics (Figure 5D). Based on Bradford’s Law, this study categorizes journals into three zones: the core zone (with ≥12 publications, consisting of 25 journals), the related zone (with 4–11 publications, 83 journals), and the marginal zone (with 1–3 publications, 394 journals), as presented in Supplementary Table 4. This journal partitioning suggests that the field of sepsis and PTM research has maintained a relatively stable development trend between 2005 and 2024. Future research can further analyze the thematic distribution and research methods of different journal clusters to gain a deeper understanding of the knowledge structure and development trends in this field.

### 3.5 Author distribution

Based on Price’s theorem, the formula for calculating the minimum number of publications for a core author in a field is m = 0.749 × √Nmax, where Nmax represents the highest number of publications by an author in that field. Applying this formula, we obtain m = 0.749 × √35. Authors with three or more publications are considered core authors. Using VOSviewer, we identified 273 core authors with three or more publications, accounting for 1,266 articles, which represent 74.3% of the total publications. This is generally in line with Price’s standard, indicating that a stable group of core authors has formed in the field of sepsis and PTM. These core authors mainly come from countries such as China and the United States. Among these prolific core authors, Lang, Charles H., ranks first with the highest number of publications and citations. His main research focuses on the repair mechanisms that regulate skeletal muscle protein balance during sepsis recovery, exploring the significant role of muscle-derived immune activation in sepsis. The top five authors by publication volume are listed in Supplementary Table 5. By organizing and studying the articles of these prolific core authors, we found that most scholars’ research on post-translational modifications primarily revolves around the protection of organs such as lungs and kidneys. Through studying specific post-translational modification changes, they aim to reduce organ dysfunction and treat sepsis by mitigating inflammatory responses in corresponding areas.

### 3.6 Analysis of cited references and cited journal

**Figure 6:**
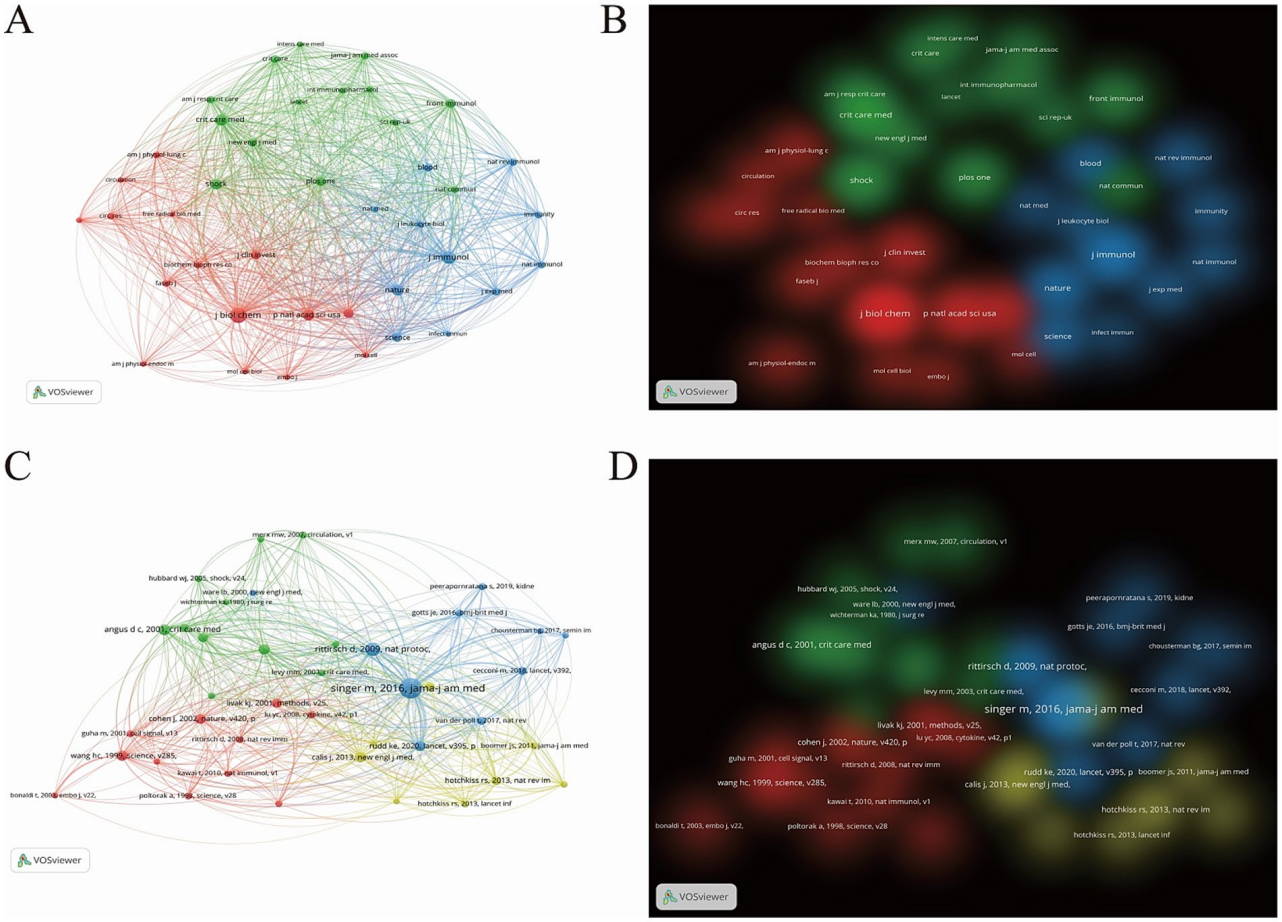
**(A)** Network visualization map of co-cited journal distribution. **(B)** Density visualization map of co-cited journal distribution. **(C)** Network visualization map of co-cited literature distribution. **(D)** Density visualization map of co-cited literature distribution.

The 1705 valid articles in this study cited references from 4,459 journals, among which 40 journals were finally selected based on a citation count exceeding 361. As shown in Figures 6A,B, the co-citation network of journals can be mainly divided into three clusters. The top three journals in terms of citation frequency are the biochemical journal “Journal Of Biological Chemistry” (cited 3,163 times, IF2024 = 4.0), the immunology journal “Journal Of Immunology” (cited 2,210 times, IF2024 = 3.6), and the critical care medicine journal “Critical Care Medicine” (cited 1723 times, IF2024 = 7.7). The red cluster is dominated by journals in the field of critical care medicine; the blue cluster focuses on immunology journals; and the green cluster consists primarily of biochemical journals. These clusters carry almost equal weight and are closely related to each other.

Furthermore, using VOSviewer software, we analyzed 62,733 cited articles and selected 40 articles with a citation count of ≥25. We then constructed a co-citation network diagram (Figures 6C,D). These highly cited articles can be categorized into four clusters. Among them, the co-cited articles in the blue and yellow clusters are concentrated between 2010 and 2020, focusing on more novel research content, while the co-cited articles in the red and green clusters are centered between 2000 and 2010. The top five most-cited articles (Supplementary Table 6), are mostly epidemiological studies related to sepsis, indicating that sepsis remains a major global health concern, and there is an urgent need to develop new diagnostic and treatment methods.

### 3.7 Keywords and research hotspots

**Figure 7:**
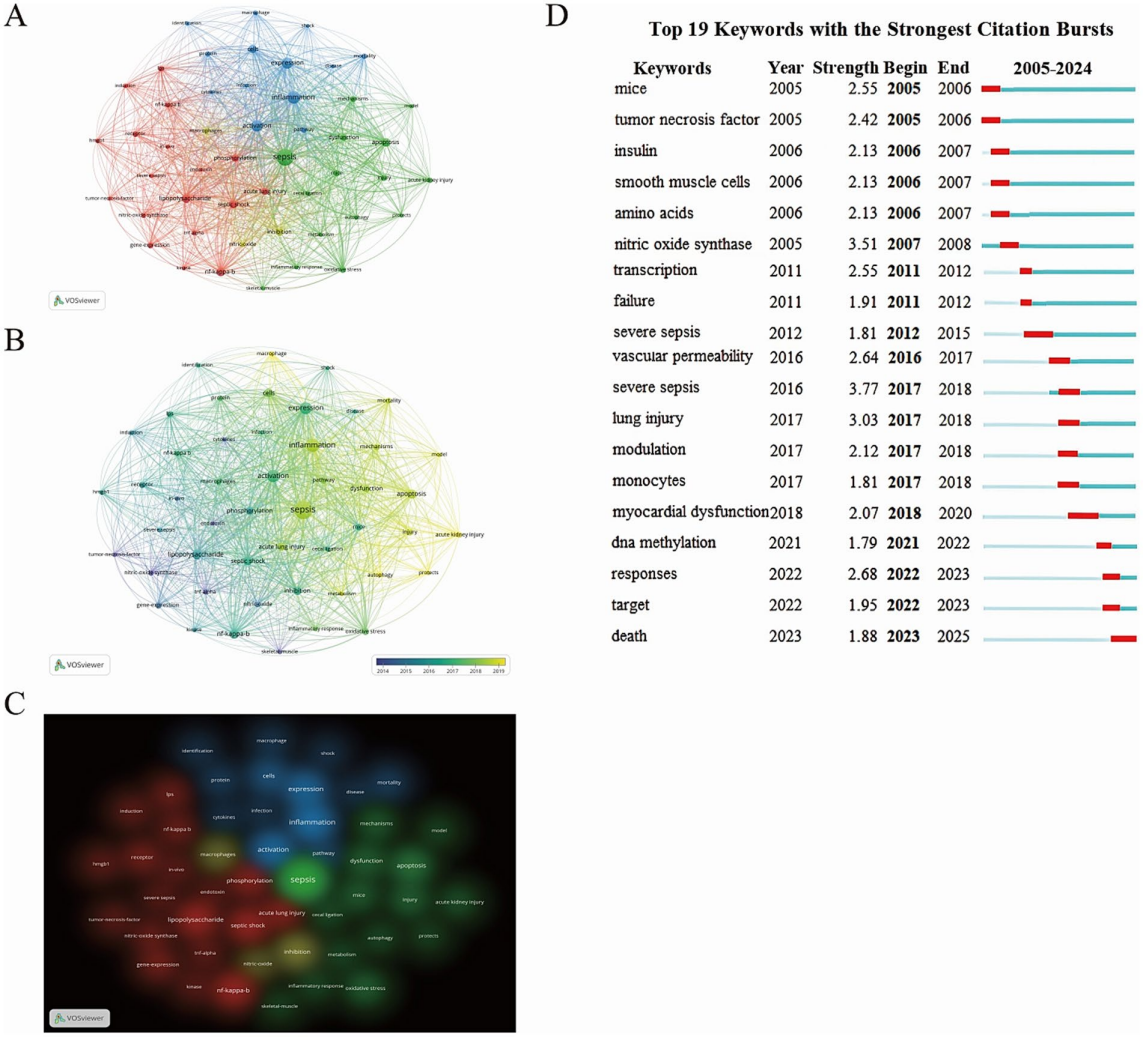
**(A)** Network visualization map of keyword distribution. **(B)** Overlay visualization map of keyword distribution (Chromatic encoding by publication year: heightened vividness signifies greater recency). **(C)** Density visualization map of Keyword Distribution. **(D)** Temporal Distribution of the Top 19 Emergent Keywords (Red markers denote years of heightened citation bursts).

Using VOSviewer, a total of 6,507 keywords were generated, and 49 high-frequency keywords with occurrences of 47 or more were selected for further analysis (Figures 7A–C). These high-frequency keywords include sepsis (*n* = 801), inflammation (*n* = 446), nf-kappa-b (*n* = 207), apoptosis (*n* = 196), and phosphorylation (*n* = 142). The cluster and density views in VOSviewer indicate that these keywords can be divided into four distinct clusters. The green cluster primarily involves sepsis, dysfunction, acute kidney injury, metabolism, etc. The red cluster mainly focuses on lipopolysaccharide, phosphorylation, nf-kappa-b, tumor necrosis factor, and so on. The blue cluster concerns activation, inflammation, gene expression, mortality, and other related topics. The yellow cluster centers around inhibition, macrophages, and nitric oxide. Furthermore, VOSviewer’s overlay visualization maps the publication years of keywords through color, visually demonstrating the evolving trends of research hotspots. The results reveal that sepsis has been a central research theme in this field since 2005, while keywords like acute kidney injury, macrophages, and autophagy have emerged as new research foci in recent years, reflecting a shift in the field’s research direction. To further explore the dynamic changes in research hotspots, this study also employed CiteSpace for keyword citation burst analysis. Figure 7D, it illustrates the keywords with the strongest citation bursts from 2005 to 2024. The red lines represent the timeframes of the keyword bursts. Specifically, the keyword “tumor necrosis factor” exhibited a high citation strength in 2005 (2.42), indicating its significance in related studies. Notably, “nitric oxide synthase” demonstrated the highest citation strength (3.51) from 2005 to 2008, highlighting the enzyme’s prominent role in relevant research. The significant increase in citation strength for keywords like “DNA methylation” and “myocardial dysfunction” marks them as new research hotspots, reflecting growing attention from researchers on sepsis-induced myocardial dysfunction and the role of DNA methylation modifications in sepsis. Collectively, these findings untangle the evolution of key research topics and hotspots in the field of sepsis, providing valuable insights for future research directions.

Quantitative analysis of the types of post-translational protein modifications covered in 1705 articles was conducted using VOSviewer (Supplementary Table 7). The results indicate that phosphorylation modification is the most widely studied in sepsis research, with the keyword appearing 142 times. This is followed by ubiquitination, methylation, acetylation, nitrosylation, and glycosylation. Research on phosphorylation modification primarily focuses on tyrosine phosphorylation in the pathophysiology of sepsis, regulation of cellular functions, and potential therapeutic targets. Notably, there is a significant concentration of publications related to ubiquitination and methylation modifications, primarily between 2019 and 2025. This suggests that these areas are currently hot topics in sepsis research. These findings not only untangle the current state of post-translational protein modification research in sepsis but also provide crucial clues for future research directions. For instance, further exploration of the specific mechanisms of ubiquitination and methylation modifications in sepsis, as well as their potential as therapeutic targets, could be fruitful. Additional research efforts can concentrate on these emerging types of post-translational modifications to untangle more details about the pathophysiology of sepsis and ultimately develop more effective treatment strategies.

## 4 Discussion

### 4.1 General information

In this study, the number of publications on PTM and sepsis has shown an overall upward trend, with a significant increase between 2019 and 2020, indicating that this research area is receiving increasing attention. This trend may be closely related to the mature application of CRISPR-Cas9 gene editing technology and the breakthrough progress of single-cell sequencing technology. Although the citation rate in this field has declined in the past 3 years, the number of papers published in the past 5 years accounts for 43.4% of the total publications, which is still a high proportion. This fully reflects the importance and continued academic influence of PTM in sepsis research. Meanwhile, this unique phenomenon of “high output but slow citation” suggests that the field is in a critical stage of transitioning from theoretical innovation to clinical application.

The journal with the most publications on PTM and sepsis is “Shock” (*n* = 77, 4.52%). This journal currently focuses on promoting and highlighting advancements in the field of protein post-translational modifications and sepsis. The “Journal of Immunology” (with an average citation of 65.75) predominantly publishes original research papers and reviews. Its primary research directions include the role of protein modifications in the pathophysiology of sepsis, protein modifications as biomarkers for sepsis diagnosis and prognosis, and potential therapeutic targets. From the author’s perspective, a stable core group of authors has formed within this discipline, mainly from countries such as China and the United States. Among these prolific core authors, Lang, Charles H. has published the most articles and is also the most cited author. His research focuses on the repair mechanisms that regulate skeletal muscle protein balance during sepsis recovery and the significant role of muscle-derived immune activation in sepsis. Many core authors approach the subject from the perspective of organ protection, providing numerous potential therapeutic targets for sepsis treatment.

The citation frequency of co-cited references can serve as an indicator for evaluating their influence within a specific research field; thus, it may be regarded as the research foundation within a given domain (16, 17). In this study, the five most frequently co-cited references were selected to illustrate the research landscape of PTM in sepsis. Among them, three articles focus on sepsis-related epidemiological studies (1, 2, 18). The most frequently cited study, published by Singer et al. (1), provides a unified treatment standard for sepsis in global medical institutions and emphasizes the crucial role of rapid and effective treatment in the early stages to improve the prognosis of sepsis patients. Rittirsch et al. (19) defined the severity level of sepsis by adjusting the position of cecal ligation, thereby establishing a standardized procedure for inducing sepsis in mice and rats, which has greatly advanced the development of molecular mechanistic studies in this field. The article published by Job Calis in the “NEW ENGLAND JOURNAL OF MEDICINE” (IF2024 = 96.2, Q1) with the highest impact factor summarizes the diagnostic criteria for sepsis, severe sepsis, and septic shock. It also elaborates on its pathophysiology (innate immunity and immunosuppression, inflammatory and anti-inflammatory mechanisms, coagulation abnormalities, organ dysfunction) and treatment methods. The article points out that despite significant progress in reducing the risk of immediate death associated with sepsis, there is still a need for newer and smarter clinical trial design and execution methods to develop new therapeutic drugs (20). In summary, the widely cited literature mainly focuses on elucidating the epidemiology, pathophysiology, and diagnostic criteria of sepsis.

### 4.2 Research hotspots and trends

In recent years, sepsis and PTMs have garnered increasing attention from scholars worldwide. Based on the analysis of keyword citation bursts and tag clouds, “sepsis” has been a persistent keyword since 2005, while terms like “acute kidney injury” and “autophagy” have emerged recently, reflecting the latest research hotspots and directions. This also indicates the depth and detail of the research. Additionally, this study analyzed keywords related to PTM, with phosphorylation being the most researched topic. Publications on ubiquitination and methylation modifications were mainly concentrated between 2019 and 2024. This trend highlights not only the growing interest in these topics within the academic community, but also suggests that they are poised to become key areas and primary avenues for further investigation.

#### 4.2.1 Sepsis-associated acute kidney injury

SA-AKI refers to AKI that occurs within 7 days of a confirmed sepsis diagnosis and meets both the Sepsis-3 sepsis diagnostic criteria and the KDIGO AKI criteria (21, 22). It encompasses both sepsis-induced AKI caused by direct damage from sepsis and AKI resulting from indirect damage (such as nephrotoxic drugs or abdominal compartment syndrome) caused by sepsis or its treatment. As the most vulnerable organ during sepsis, the kidney is affected in up to 45–70% of patients with sepsis, and SA-AKI is associated with higher ICU and in-hospital mortality rates than either sepsis or AKI alone (23). Therefore, SA-AKI indicates a poorer prognosis than any isolated syndrome and is correlated with increased long-term disability rates and reduced quality of life in both adults and children (24, 25). The pathophysiological mechanisms of SA-AKI are complex, and progress in treatment options has been limited. However, the development of targeted PTMs for SA-AKI is actively underway, with studies such as the investigation of the class IIa HDAC inhibitor TMP195, which may help restore cellular function and reduce the severity of AKI (12). Additionally, inhibitors targeting specific phosphorylation or ubiquitination enzymes should be a focus of future research in SA-AKI. By detecting the presence of specific PTMs, the development of new biomarkers for early diagnosis and prognostic evaluation of SA-AKI can assist clinicians in better understanding disease progression and formulating individualized treatment plans. For instance, phosphorylated MYL12B is considered a potential biomarker in the plasma of patients with SA-AKI (26). Gene therapy, utilizing gene-editing techniques like CRISPR-Cas9 to target enzymes related to PTMs, is seen as one of the most promising innovative therapies. While there have been reports of using gene-editing techniques in the treatment of sepsis, further research is needed considering the limitations of targeted genes and the instability of CRISPR/Cas9 technology in sepsis treatment (27). Finally, due to the high heterogeneity of SA-AKI, individualized treatment plans based on patients’ protein modification profiles and specific PTM characteristics can enhance treatment success rates.

#### 4.2.2 Autophagy

Autophagy is a process through which cells degrade and recycle proteins and organelles to maintain intracellular homeostasis. Typically, autophagy plays a protective role in cells, yet disruption of the autophagy mechanism or excessive autophagy flux often leads to cell death (28). Autophagy can counteract sepsis by regulating both innate and adaptive immune processes, with different regulatory mechanisms in various immune cells. On one hand, autophagy can help alleviate inflammatory responses and protect organs from further damage; on the other hand, in certain cases, excessive or insufficient autophagy may exacerbate the condition (29). Both the tumor suppressor gene P53 and Belin-1 are autophagy regulators, and upregulation of autophagy can alleviate sepsis-induced acute kidney injury. However, during sepsis, there is no significant change in the expression of p53 protein, but there is an increase in the translocation of p53 from the nucleus to the cytoplasm and an increase in p53 acetylation. Activation of the deacetylase Sirtuin 1 (Sirt1) or mutation of the acetylated lysine site in p53 leads to p53 deacetylation, which induces RTEC autophagy and alleviates SA-AKI (30). Additionally, SIRT1 can mediate the deacetylation of Beclin1 at sites K430 and K437, and upregulation of Beclin1 deacetylation induces autophagy, exerting a protective effect on SA-AKI (31). Similarly, in sepsis-induced cardiac insufficiency, relief can be observed through Sirt1-mediated Beclin-1 deacetylation and autophagy (32). Drug induction of autophagy via SIRT1-mediated Beclin-1 deacetylation may represent a promising therapeutic approach for sepsis treatment in the future. Furthermore, SIRT1 deficiency promotes excessive mtROS generation and mtDNA leakage into the cytoplasm by impairing late endosome-mediated mitochondrial autophagy, leading to enhanced activation of NLRP3 and STING, which results in pulmonary endothelial dysfunction in sepsis (33). There is a close interaction between the NLRP3 inflammasome and autophagy, jointly regulating cellular homeostasis and inflammatory responses (34). As an antidiabetic drug, Biguanide (BF) upregulates autophagy and Nrf2 protein levels through an AMPK-dependent pathway, thereby inhibiting NLRP3-mediated pyroptosis in sepsis-induced ALI (35). These changes in pathways point to new therapeutic targets for the treatment of sepsis-induced ALI. In sepsis-associated encephalopathy (SAE), aquaporin-4 (AQP4) knockout alleviates learning and memory impairments in an SAE mouse model by activating autophagy, inhibiting neuroinflammation, and exerting neuroprotective effects by downregulating the Nav1.6 channel in astrocytes (36). However, excessive autophagy activation can also exacerbate sepsis. For instance, in sepsis-induced muscle loss, amino acid supplementation is necessary to antagonize the activation of skeletal muscle autophagy signals and prevent sepsis-induced muscle protein degradation (37). Some studies have shown that autophagy activation exacerbates inflammatory responses. In sepsis animal models treated with autophagy activators, the concentrations of inflammatory cytokines such as IL-1β and TNF-*α* in the serum can increase several times or even more compared to the untreated group. Simultaneously, a significant increase in immune cell infiltration at the site of inflammation and a shift in the polarization state of immune cells towards a pro-inflammatory direction are observed. In studies related to cell death pathways, it has been found that the expression of pyroptosis-related proteins is significantly higher in the excessive autophagy activation group compared to the normal control group, accompanied by more severe tissue damage and inflammatory manifestations (38, 39).

Despite numerous studies demonstrating the importance of autophagy in sepsis, the specific regulatory mechanisms of autophagy still require further exploration. Future research should focus on how to more precisely control the level of autophagy to achieve the best therapeutic effect, and more clinical trials are needed to verify the effectiveness and safety of existing animal experiment results (40, 41).

#### 4.2.3 Ubiquitination

Ubiquitination is a widespread and biologically significant post-translational modification process of proteins within cells. Essentially, ubiquitination refers to the process of covalently attaching the small protein ubiquitin to a target protein (42). Ubiquitin is a highly conserved polypeptide chain consisting of 76 amino acids, prevalent in eukaryotes with a highly similar sequence. During ubiquitination, the ubiquitin molecule first needs to be activated. This activation step is typically carried out by a ubiquitin-activating enzyme (E1), which activates the ubiquitin molecule by consuming ATP to form an intermediate connected by a high-energy thioester bond (43). Subsequently, the activated ubiquitin is transferred to a ubiquitin-conjugating enzyme (E2), forming an E2-ubiquitin complex. Finally, under the action of a ubiquitin ligase (E3), ubiquitin is transferred from the E2-ubiquitin complex and covalently attached to specific lysine residues of the target protein, thus completing the ubiquitination modification process. This process can be monoubiquitination, where only one ubiquitin molecule is attached to the target protein, or polyubiquitination, where multiple ubiquitin molecules are connected in sequence to form a ubiquitin chain attached to the target protein. Different types of ubiquitin chain linkages (such as through different lysine residues) can also confer different fates and functions to the target protein (44, 45).

Ubiquitination, as a crucial post-translational modification of proteins, plays a significant role in various pathophysiological processes of sepsis. In the early stages of sepsis, the inflammatory response intensifies, and inflammatory cytokines become pivotal in the pathophysiology of the condition. Ubiquitin-specific peptidase 18 (USP18) is a deubiquitinating enzyme. Macrophages deficient in USP18 exhibit enhanced activation of NF-κB, p38, and ERK, accompanied by increased ubiquitination of transforming growth factor-*β*-activated kinase 1 (TAK1). Mice lacking USP18 in their macrophages show reduced survival rates and elevated levels of proinflammatory cytokines such as IL-6 and IL-1β in their serum (46). Similarly, USP19 interacts with TAK1 in a TNF-*α* or IL-1β-dependent manner, specifically dissociating K63- and K27-linked polyubiquitin chains from TAK1. This interaction impairs TAK1 activity and disrupts the TAK1-TAB2/3 complex. Compared to wild-type littermates, Usp19−/− mice produce higher levels of inflammatory cytokines and are more susceptible to TNF-α and IL-1β-induced septic death (47). The ability of deubiquitinating enzymes to modulate the activity of TAK1, a key player in inflammatory signaling pathways, suggests that ubiquitination-related enzymes can influence the early inflammatory response in sepsis by regulating the activity of critical molecules in these pathways.

Sepsis patients often exhibit severe hemostatic disorders and fibrinolysis impairment, manifested by systemic thrombin generation, compromised anticoagulation activity, and fibrinolysis inhibition (48). Ubiquitination also plays a role in the regulation of coagulation-related proteins. The NLR family pyrin domain-containing-3 (NLRP3) inflammasome plays a crucial role in the host’s defense against microbial pathogens, and its dysregulation may trigger coagulation cascades. YOD1, a deubiquitinating enzyme, interacts with NLRP3 and specifically inhibits its expression and the activation of the NLRP3 inflammasome. Defects in YOD1 expression enhance the activation of the NLRP3 inflammasome and coagulation both *in vitro* and *in vivo* (49). Although it remains unclear which specific coagulation-related proteins undergo ubiquitination modifications that are critical in sepsis-induced coagulation disorders, modulating the ubiquitination status of key molecules in coagulation-related signaling pathways may alter the initiation, propagation, or termination of the coagulation response (50).

Endothelial dysfunction plays a pivotal role in the pathophysiology of sepsis and organ failure. The surface of endothelial cells harbors a variety of receptors and signaling molecules, and the ubiquitination status of these molecules may affect their binding affinity to ligands and the efficiency of intracellular signal transduction. In the septic environment, pathogens and their products, inflammatory mediators, and other factors may alter the ubiquitination pattern of molecules associated with endothelial cells. This alteration can lead to endothelial dysfunction, further exacerbating the severity of sepsis and the risk of organ damage (51). YAP (Yes-associated protein), the primary transcriptional co-activator of the Hippo pathway, interacts with the E3 ubiquitin-protein ligase TLR (Toll-like receptor) signaling adaptor protein TRAF6 (Tumor Necrosis Factor Receptor-Associated Factor 6) in the CLP mouse model. This interaction ubiquitinates TRAF6, promoting its degradation and modification, thereby inhibiting NF-κB activation and suppressing vascular inflammation (52). Endothelial activation plays a crucial role in the pathophysiology of sepsis-induced acute lung injury. TRIM47, an E3 ubiquitin ligase belonging to the tripartite motif-containing protein family, is highly expressed in vascular endothelial cells. It can enhance the K63-linked ubiquitination of TRAF2, promoting LPS-induced lung inflammation and acute lung injury. This, in turn, activates the NF-κB and MAPK signaling pathways, triggering inflammatory responses in endothelial cells (53). Notch signaling is essential for regulating the function of vascular endothelial cells. LPS reduces the stability of the intracellular domain (NICD) of Notch1 by inhibiting the expression of the deubiquitinating enzyme ubiquitin-specific protease 8 (USP8), leading to vascular endothelial dysfunction (54).

Mitochondria play a crucial role in cellular energy metabolism. During sepsis, mitochondrial function often undergoes alterations. Ubiquitination may be involved in the regulation of mitochondrial-related proteins. For instance, mitochondrial STAT3 can trigger fatty acid oxidation by inducing the stabilization of CPT1a mediated by USP50 in macrophages, exacerbating LPS-induced sepsis (55). PINK1 (PTEN-induced putative kinase 1) is a mitochondrial-targeted serine/threonine kinase that serves as a critical regulator for maintaining mitochondrial function. Upon mitochondrial damage or depolarization, PINK1 accumulates on the outer mitochondrial membrane (OMM) and recruits the E3 ubiquitin ligase Parkin. Subsequently, Parkin ubiquitinates OMM proteins to tag damaged mitochondria for autophagic degradation, thereby eliminating impaired mitochondria and preserving mitochondrial population health (56, 57). PINK1 plays a pivotal role in sustaining mitochondrial homeostasis and mitigating sepsis-associated injury. During sepsis, PINK1 knockout suppresses Parkin-dependent mitophagy while enhancing dynamin-related protein 1 (Drp1)-mediated mitochondrial fission, ultimately leading to dendritic cell dysfunction and exacerbated immunosuppression (58). In murine hearts during sepsis, reduced PINK1 expression impairs mitochondrial Ca^2+^ efflux in cardiac cells, resulting in mitochondrial calcium overload and cardiomyocyte injury. Exosomes derived from human mesenchymal stem cells (huMSC-exo) carry PINK1 mRNA, which can be transferred to recipient cardiac cells to upregulate PINK1 expression, ameliorate mitochondrial calcium efflux dysfunction, and attenuate cardiomyocyte damage (59). Furthermore, PINK1 and PARK2 (parkin RBR E3 ubiquitin protein ligase) exhibit neuroimmunoprotective effects in sepsis. The PINK1/PARK2-dependent neuroimmune pathway modulates peripheral inflammation by regulating dopamine release, HIF1A and NLRP3 inflammasome activation, and HMGB1 secretion in murine models. This neuroimmune axis coordinates metabolic and inflammatory responses, contributing to hyperlactatemia, tissue injury, and even organ dysfunction (60). Overexpression of circRNA itchy E3 ubiquitin protein ligase (circ-ITCH) can improve renal function in SA-AKI mice and regulate oxidative stress and mitochondrial dysfunction in sepsis-induced AKI through the miR-214-3p/ABCA1 pathway, further enriching the pathophysiology of SA-AKI (61). Therefore, ubiquitination affects the functional status of mitochondrial membrane transport proteins and enzymes involved in the oxidative phosphorylation process through ubiquitin modification, thereby influencing mitochondrial functions in sepsis, such as energy production and reactive oxygen species generation. This ultimately impacts the progression of sepsis and organ function. However, specific ubiquitination targets and regulatory mechanisms require further investigation. Future research directions can focus on identifying key ubiquitination modification target proteins and changes in their modification patterns in different stages of sepsis and various organ tissues. Additionally, exploring the synergistic or antagonistic effects of ubiquitination and other post-translational modifications (such as phosphorylation and methylation) in the pathophysiology of sepsis could provide new insights and methods for the treatment and intervention of sepsis.

#### 4.2.4 Methylation

DNA methylation, a profound epigenetic modification, primarily refers to the chemical modification process where a methyl group (typically derived from S-adenosylmethionine) is added to specific DNA regions or loci under the catalytic action of DNA methyltransferases (62). Besides DNA methylation, protein methylation also occurs within cells. Protein methylation involves the addition of a methyl group to specific amino acid residues of a protein. Various amino acid residues, such as lysine (K) and arginine (R), are susceptible to methylation modification (63).

Sepsis is often accompanied by an uncontrollable inflammatory response in its early stages, and methylation plays a role in regulating inflammation-related signaling pathways. For instance, the toll-like receptor (TLR) signaling pathway is crucial in initiating and regulating inflammatory responses. Protein arginine methyltransferase 2 (PRMT2) can enhance the production of interferon-*β* (IFN-β) through the TLR4/IRF3 signaling pathway, further balancing the inflammatory response. NF-κB is widely recognized as a master regulator in the process of pro-inflammatory transcription. There is growing evidence highlighting the importance of histone methylation in NF-κB-dependent inflammatory responses (64). The long-term inflammatory damage and impaired wound healing caused by sepsis are coordinated by downregulating the methyltransferase MLL1 and H3K4me3 at inflammatory gene loci of NF-κB in the bone marrow, leading to persistent impairment of peripheral macrophage function (65). MicroRNAs (miRs) play a key pathogenic role in sepsis-induced acute kidney injury (AKI). In LPS-treated HK2 cells, miR-93-5p and histone H3 Lys27 trimethylation (H3K27me3) are downregulated, while KDM6B is upregulated. Further studies have found that silencing KDM6B induces H3K27me3, inhibits the activation of TNF-*α*, thereby attenuating LPS-induced damage in HK2 cells. *In vivo* studies have also found that EPC-derived extracellular vesicles (EVs) containing miR-93-5p alleviate multi-organ damage, vascular leakage, inflammation, and apoptosis in septic mice (66). These studies collectively demonstrate the significant impact of methylation in inflammatory regulatory pathways.

Sepsis patients often present with lymphopenia, in which methylation plays a role. Mechanistically, methylation may affect the development, differentiation, and activation of lymphocytes. For instance, by altering the methylation status of lymphocyte-related genes, it can influence their normal immune function, resulting in a decreased immune defense capability during sepsis. This makes the body more susceptible to pathogen invasion and is associated with a persistent immunosuppressive state (67). In a study on neonatal sepsis, changes in methylation levels were observed in genomic regions involved in inflammatory pathways related to innate and adaptive immune responses. Methylation altered the expression patterns of immune-related genes, impairing the immune system’s ability to normally respond to infections. This leaves the body in a state of immunocompromise, which is detrimental to the healthy growth of newborns (68).

Biomarkers, as objective indicators for evaluating patients’ conditions, hold significant value in disease diagnosis, treatment, and prognosis evaluation. Due to the ease of DNA isolation, DNA methylation stands out as a highly potential biomarker (69). Binnie et al. (70) investigated the genome-wide DNA methylation profiles of adult septic patients, identifying 668 differentially methylated regions corresponding to 443 genes. Functional analysis revealed enrichment in antigen processing and presentation, methyltransferase activity, cell adhesion, and cell junction. A weighted gene co-expression network analysis untangled DNA co-methylation modules associated with clinical features, including disease severity, the need for vasopressors, and hospital stay duration (70). Furthermore, dysregulation of RNA methylation genes (m1A, m5C, m6Am, m7G, and *Ψ*) is closely linked to the pathophysiology of sepsis and could serve as novel diagnostic biomarkers and potential therapeutic targets (71).

Methylation modifiers also hold significant potential in the treatment of sepsis. The antiarrhythmic drug procainamide, a non-nucleoside inhibitor of DNA Methyltransferase 1 (DNMT1), is used to reduce DNA hypermethylation. In LPS-induced rat models, procainamide treatment not only inhibits the overexpression of DNMT1 but also reduces the excessive production of IL-6 in rats with rhabdomyolysis, thereby improving renal function (72). In an endotoxin-induced rat model, DNMT1 inhibitors lowered DNMT1 and 5-methylcytosine levels, reducing IL27RA methylation in the lungs of endotoxin-treated rats. This, in turn, improved neutrophil infiltration and superoxide production in the lungs (73). It’s worth noting that epigenetic modifiers are mostly used for early intervention, suggesting that these agents may be more effective during the initial stages of the inflammatory response. Currently, drugs targeting DNMT1 inhibitors face challenges related to specificity when applied to sepsis treatment. Some existing DNMT1 inhibitors may not only affect DNMT1 but also impact other related enzymes or normal physiological processes within cells, potentially causing undesirable side effects. The high consistency of sepsis leads to significant variations in genetic backgrounds, baseline health statuses, and immune states among different individuals during sepsis onset. Such individual differences can significantly alter the effects of epigenetic modifiers and the responses to related treatments among patients.

### 4.3 Strengths and limitations of the study

This study conducted the first comprehensive visual analysis of post-translational modifications and sepsis research using bibliometric methods, revealing research hotspots, core author groups, and the distribution of high-impact journals in this field from a macro perspective. It provides a new viewpoint for further understanding this complex domain. Simultaneously, the analysis of cited references and keywords will assist researchers in comprehending popular studies in this area, as well as identifying potential research hotspots and future directions. Inevitably, this study has some limitations. Firstly, bibliometrics can only present the research situation at the macro level and cannot delve into the detailed mechanisms of specific PTMs at the cellular and molecular levels in sepsis. Secondly, the research publications in this paper are solely from WOS, without considering CNKI and other databases. Additionally, the publication language is limited to English, and important publications in other languages may be overlooked. Despite these limitations, employing visualization methods to identify the current state, hotspots, and trends in this field remains highly valuable.

## 5 Conclusion

This study, through bibliometric analysis, systematically untangles the research dynamics and core trends in the field of PTM related to sepsis for the first time. China dominates in terms of publication volume, while research from countries like the United States demonstrates higher citation impact, highlighting the importance of international collaboration. Phosphorylation, ubiquitination, and methylation are current research hotspots, with emerging directions such as SA-AKI, autophagy regulation, and mitochondrial dysfunction taking center stage. In the future, it is necessary to deepen mechanistic studies on novel PTM, promote the development of targeted therapeutic strategies, and integrate resources through cross-border cooperation to accelerate the clinical translation of precision medicine for sepsis.

## Data Availability

The original contributions presented in the study are included in the article/Supplementary material, further inquiries can be directed to the corresponding authors.
