# Supplementary material for: New trends and hotspots in sepsis-related protein post-translational modification: a bibliometric and visual analysis
Source: Front Med (Lausanne). 2025 Jul 22;12:1606786. doi: 10.3389/fmed.2025.1606786 (PMC12321805; doi:10.3389/fmed.2025.1606786)
Supplement: Supplementary file 1 [file Table_1.docx]

**Table 1.The top 5 countries with most publications on sepsis-related protein post-translational modifications**

| Rank | Country | Publication Count | Citations | Average Citation Count |
| --- | --- | --- | --- | --- |
| 1 | China | 830 | 20788 | 25.046 |
| 2 | USA | 499 | 29075 | 58.267 |
| 3 | South Korea | 104 | 4607 | 44.298 |
| 4 | Germany | 104 | 4089 | 39.317 |
| 5 | Japan | 72 | 2006 | 27.861 |
