# Supplementary material for: New trends and hotspots in sepsis-related protein post-translational modification: a bibliometric and visual analysis
Source: Front Med (Lausanne). 2025 Jul 22;12:1606786. doi: 10.3389/fmed.2025.1606786 (PMC12321805; doi:10.3389/fmed.2025.1606786)
Supplement: Supplementary file 2 [file Table_2.docx]

**Table 2.The top 10 institutions with most publications on sepsis-related protein post-translational modifications**

| Rank | Institution | Publication Count | Citations | Average Citation Count |
| --- | --- | --- | --- | --- |
| 1 | shanghai jiao tong univ | 51 | 1072 | 21.02 |
| 2 | southern med univ | 49 | 985 | 20.10 |
| 3 | cent south univ | 43 | 992 | 23.07 |
| 4 | china med univ | 36 | 1007 | 27.97 |
| 5 | fudan univ | 34 | 1151 | 33.85 |
| 6 | wuhan univ | 34 | 631 | 18.56 |
| 7 | nanjing med univ | 32 | 992 | 31.00 |
| 8 | univ pittsburgh | 30 | 3296 | 109.87 |
| 9 | tongji univ | 28 | 941 | 33.61 |
| 10 | zhejiang univ | 27 | 951 | 35.22 |
