# Supplementary material for: New trends and hotspots in sepsis-related protein post-translational modification: a bibliometric and visual analysis
Source: Front Med (Lausanne). 2025 Jul 22;12:1606786. doi: 10.3389/fmed.2025.1606786 (PMC12321805; doi:10.3389/fmed.2025.1606786)
Supplement: Supplementary file 3 [file Table_3.docx]

**Table 3.The top 10 sources with most publications on sepsis-related protein post-translational modifications**

| Rank | Source | Publication Count | Citations | Average Citation Count |
| --- | --- | --- | --- | --- |
| 1 | Shock | 77 | 1717 | 22.299 |
| 2 | International immunopharmacology | 65 | 1094 | 16.831 |
| 3 | Frontiers in immunology | 55 | 960 | 17.455 |
| 4 | Plos one | 47 | 1452 | 30.894 |
| 5 | Journal of immunology | 40 | 2630 | 65.75 |
| 6 | Critical care medicin | 32 | 1739 | 54.344 |
| 7 | Journal of biological chemistry | 30 | 1869 | 62.3 |
| 8 | International journal of molecular sciences | 29 | 304 | 10.483 |
| 9 | Scientific reports | 28 | 929 | 33.179 |
| 10 | inflammation | 19 | 355 | 18.684 |
