# Supplementary material for: New trends and hotspots in sepsis-related protein post-translational modification: a bibliometric and visual analysis
Source: Front Med (Lausanne). 2025 Jul 22;12:1606786. doi: 10.3389/fmed.2025.1606786 (PMC12321805; doi:10.3389/fmed.2025.1606786)
Supplement: Supplementary file 4 [file Table_4.docx]

**Table 4.The number of journals and publications in each zone**

| Zone | Publications per Journal | The number of journals | Total Publication Count |
| --- | --- | --- | --- |
| The first zone | ≥12 | 25 | 574 |
| The second zone | 4-11 | 83 | 612 |
| The third zone | 1-3 | 394 | 549 |
