# Supplementary material for: New trends and hotspots in sepsis-related protein post-translational modification: a bibliometric and visual analysis
Source: Front Med (Lausanne). 2025 Jul 22;12:1606786. doi: 10.3389/fmed.2025.1606786 (PMC12321805; doi:10.3389/fmed.2025.1606786)
Supplement: Supplementary file 5 [file Table_5.docx]

**Table 5.The top 5 authors with most publications on sepsis-related PTM**

| Rank | Author | Count | Citations | Average Citation Count | Main Research Subjects | Main Types of PTM |
| --- | --- | --- | --- | --- | --- | --- |
| 1 | Lang,charles h. | 20 | 797 | 39.85 | Muscle | Phosphorylation |
| 2 | Huang,qiaobing | 13 | 312 | 24 | Lung | Ubiquitination,Acetylation |
| 3 | Wang,yi | 13 | 277 | 21.208 | Lung,Kidney | Ubiquitination,Phosphorylation |
| 4 | Bae,jong-sup | 12 | 259 | 21.583 | Blood Vessel | Acetylation,Phosphorylation |
| 5 | Chen,jie | 12 | 234 | 19.5 | Lung,Liver | Phosphorylation |
