# Supplementary material for: New trends and hotspots in sepsis-related protein post-translational modification: a bibliometric and visual analysis
Source: Front Med (Lausanne). 2025 Jul 22;12:1606786. doi: 10.3389/fmed.2025.1606786 (PMC12321805; doi:10.3389/fmed.2025.1606786)
Supplement: Supplementary file 6 [file Table_6.docx]

**Table 6.The top 5 cited references with most Citations on sepsis-related PTM**

| Rank | Reference | Author | Year | Citations |
| --- | --- | --- | --- | --- |
| 1 | The Third International Consensus Definitions for Sepsis and Septic Shock (Sepsis-3) | [Mervyn Singer](https://pubmed.ncbi.nlm.nih.gov/?sort=date&term=Singer+M&cauthor_id=26903338) | 2016 | 271 |
| 2 | Immunodesign of experimental sepsis by cecal ligation and puncture | [Daniel Rittirsch](https://pubmed.ncbi.nlm.nih.gov/?sort=date&term=Rittirsch+D&cauthor_id=19131954) | 2009 | 118 |
| 3 | Epidemiology of severe sepsis in the United States: analysis of incidence, outcome, and associated costs of care | [D C Angus](https://pubmed.ncbi.nlm.nih.gov/?sort=date&term=Angus+DC&cauthor_id=11445675) | 2001 | 77 |
| 4 | Severe sepsis and septic shock | [Job Calis](https://pubmed.ncbi.nlm.nih.gov/?sort=date&term=Calis+J&cauthor_id=24256391) | 2013 | 72 |
| 5 | Global, regional, and national sepsis incidence and mortality, 1990-2017: analysis for the Global Burden of Disease Study | [Kristina E Rudd](https://pubmed.ncbi.nlm.nih.gov/?sort=date&term=Rudd+KE&cauthor_id=31954465) | 2020 | 70 |
