# Supplementary material for: New trends and hotspots in sepsis-related protein post-translational modification: a bibliometric and visual analysis
Source: Front Med (Lausanne). 2025 Jul 22;12:1606786. doi: 10.3389/fmed.2025.1606786 (PMC12321805; doi:10.3389/fmed.2025.1606786)
Supplement: Supplementary file 7 [file Table_7.docx]

**Table 7.Ranking of protein post-translational modification types by occurrence frequency**

| Rank | Types of protein Post-Translational  Modifications | Frequency |
| --- | --- | --- |
| 1 | Phosphorylation | 142 |
| 2 | Ubiquitination | 42 |
| 3 | Methylation | 37 |
| 4 | Acetylation | 31 |
| 6 | Nitrosation | 13 |
| 7 | Glycosylation | 11 |
